# Effects of Bariatric Surgery in Obese Patients With Hypertension: The GATEWAY Randomized Trial (Gastric Bypass to Treat Obese Patients With Steady Hypertension)

**DOI:** 10.1161/CIRCULATIONAHA.117.032130

**Published:** 2019-10-08

**Authors:** Carlos Aurelio Schiavon, Angela Cristine Bersch-Ferreira, Eliana Vieira Santucci, Juliana Dantas Oliveira, Camila Ragne Torreglosa, Priscila Torres Bueno, Julia Caldas Frayha, Renato Nakagawa Santos, Lucas Petri Damiani, Patricia Malvina Noujaim, Helio Halpern, Frederico L.J. Monteiro, Ricardo Vitor Cohen, Carlos H. Uchoa, Marcio Gonçalves de Souza, Celso Amodeo, Luiz Bortolotto, Dimas Ikeoka, Luciano F. Drager, Alexandre Biasi Cavalcanti, Otavio Berwanger

**Affiliations:** 1 Research Institute (C.A.S., A.C.B.-F., E.V.S., J.D.O., C.R.T., P.T.B., J.C.F., R.N.F., L.P.D., A.B.C., O.B.); 2 Surgical Center (P.M.N., H.H., F.L.J.M.); 3 Intensive Unit (D.I.), Heart Hospital, São Paulo, Brazil.; 4 Oswaldo Cruz German Hospital, São Paulo, Brazil (R.V.C.).; 5 Heart Institute, Hypertension Unit, São Paulo, Brazil (C.H.U., L.B., L.F.D.).; 6 Department of Hypertension, Dante Pazzanese Institute of Cardiology, São Paulo, Brazil (M.G.d.S., C.A.).

**Keywords:** bariatric surgery, hypertension, obesity

## Abstract

Recent research efforts on bariatric surgery have focused on metabolic and diabetes mellitus resolution. Randomized trials designed to assess the impact of bariatric surgery in patients with obesity and hypertension are needed.

**Methods:**

In this randomized, single-center, nonblinded trial, we included patients with hypertension (using ≥2 medications at maximum doses or >2 at moderate doses) and a body mass index between 30.0 and 39.9 kg/m^2^. Patients were randomized to Roux-en-Y gastric bypass plus medical therapy or medical therapy alone. The primary end point was reduction of ≥30% of the total number of antihypertensive medications while maintaining systolic and diastolic blood pressure <140 mm Hg and 90 mm Hg, respectively, at 12 months.

**Results:**

We included 100 patients (76% female, mean age 43.8±9.2 years, mean body mass index 36.9±2.7 kg/m2), and 96% completed follow-up. Reduction of ≥30% of the total number of antihypertensive medications while maintaining controlled blood pressure occurred in 41 of 49 patients from the gastric bypass group (83.7%) compared with 6 of 47 patients (12.8%) from the control group with a rate ratio of 6.6 (95% confidence interval, 3.1–14.0; *P*<0.001). Remission of hypertension was present in 25 of 49 (51%) and 22 of 48 (45.8%) patients randomized to gastric bypass, considering office and 24-hour ambulatory blood pressure monitoring, respectively, whereas no patient submitted to medical therapy was free of antihypertensive drugs at 12 months. A post hoc analysis for the primary end point considering the SPRINT (Systolic Blood Pressure Intervention Trial) target reached consistent results, with a rate ratio of 3.8 (95% confidence interval, 1.4–10.6; *P*=0.005). Eleven patients (22.4%) from the gastric bypass group and none in the control group were able to achieve SPRINT levels without antihypertensives. Waist circumference, body mass index, fasting plasma glucose, glycohemoglobin, low-density lipoprotein cholesterol, triglycerides, high-sensitivity C-reactive protein, and 10-year Framingham risk score were lower in the gastric bypass than in the control group.

**Conclusions:**

Bariatric surgery represents an effective strategy for blood pressure control in a broad population of patients with obesity and hypertension.

**Clinical Trial Registration:**

URL: https://clinicaltrials.gov. Unique identifier: NCT01784848.

## Abstract 2

Supplemental Digital Content is available in the text.

**Clinical Perspective**

**What Is New?**

- The current body of evidence on the improvement of hypertension after bariatric surgery is reported from studies designed for a different primary end point, restricted to patients with diabetes mellitus or from observational studies.
- In this trial, 100 patients with obesity and hypertension (the majority of whom did not have diabetes mellitus) were randomized to gastric bypass or medical therapy alone.
- Patients randomized to gastric bypass were 6 times more likely to reduce ≥30% of the total number of antihypertensive medications while maintaining controlled blood pressure levels.
- In addition, 51% of the patients submitted to gastric bypass showed remission of hypertension.

**What Are the Clinical Implications?**

- Bariatric surgery represents an effective strategy for reducing antihypertensive drugs in patients with obesity and hypertension.
- Given the morbidity of surgery, our results do not imply that all patients with obesity and hypertension should be submitted to bariatric surgery.
- Treatment of hypertensive patients with obesity has multiple barriers, including nonadherence to long-term multiple antihypertensive drugs. Thus, gastric bypass represents 1 extra option to help achieve blood pressure control.
- Taken together with the improvement of the metabolic and inflammatory profile, such effects have the potential to reduce major cardiovascular events.

Given the current high prevalence of obesity,^1^ an increasing number of hypertension cases occur in people with excess weight.^2^ Several patients with hypertension require >2 medications to achieve blood pressure control,^3^ which creates challenges for long-term adherence to treatment, and it is well documented that hypertension is poorly controlled in patients with obesity.^4^

Bariatric surgery represents the most effective method to treat obesity.^5,6^ Although much recent research efforts have focused on metabolic improvement and diabetes mellitus resolution, previous studies suggest that a significant percentage of patients with coexisting obesity and hypertension are able to reduce or even discontinue their antihypertensive medications after bariatric surgery.^7–10^ Because blood pressure control was not the primary focus of these studies and most randomized trials were restricted to patients with diabetes mellitus, the effects of bariatric surgery in a broader population of patients with obesity and hypertension remain uncertain. Thus, we designed the GATEWAY trial (Gastric Bypass to Treat Obese Patients With Steady Hypertension) to assess the impact of bariatric surgery on hypertension improvement in patients with obesity.

## Methods

We will share the database containing unidentified individual participant data, data dictionary documentation, statistical analysis plan, and analysis code. Beginning 6 months and ending 24 months after article publication, the trial steering committee will evaluate proposals of studies accompanied by a statistical analysis plan and may grant access to the data for approved proposals. After 24 months, the database and accompanying documents will be publicly available in an institutional data repository.^11^

### Study Design and Oversight

The study design was published previously.^12^ Briefly, this is a randomized, nonblinded, single-center, clinical trial. The follow-up period for the primary end point is 12 months, but patients are scheduled for a 5-year extension study. The Research Ethics Board at the Heart Hospital approved the protocol. All patients provided written informed consent.

The trial was coordinated by the Research Institute at the Heart Hospital. Ethicon Inc provided unrestricted funding for this investigator-initiated trial. All authors had full and independent access to all data and vouch for the integrity and accuracy of the analysis.

### Patients

We included patients 18 to 65 years of age with hypertension,^13^ with a body mass index (BMI) ranging from 30.0 to 39.9 kg/m^2^, and treated with ≥2antihypertensive drugs at maximum doses or >2 drugs at moderate doses (Table I in the online-only Data Supplement).^14^ Exclusion criteria were systolic blood pressure ≥180 mm Hg or diastolic blood pressure ≥120 mm Hg; cardiovascular disease (myocardial infarction or stroke within 6 months, angina, coronary revascularization, heart failure); severe psychiatric disorders because of increased risk of low compliance with the study procedures; chronic kidney disease (diabetic nephropathy or glomerular filtration rate <30 mL/min); secondary hypertension, except because of sleep apnea; peripheral arterial disease; atrophic gastritis; type 1diabetes mellitus, latent autoimmune diabetes of adults, or type 2 diabetes mellitus with glycohemoglobin >7.0%; alcoholism or use of illicit drugs; current smoking; previous abdominal surgery; severe hepatic diseases; pregnancy or women of childbearing age not using effective contraceptive methods; cancer in the past 5 years; use of immunosuppressive drugs, chemotherapy, or radiotherapy; or inability to understand or adhere to study procedures (detailed criteria for eligibility are provided in Box I in the online-only Data Supplement).

### Randomization

Subjects were randomized (1:1) to either gastric bypass combined with medical therapy or medical therapy alone. Randomization was performed through a 24-hour central web-based automated system.

### Treatments

Medical therapy was standardized for all patients based on office blood pressure. (The detailed method of office blood pressure measurement is described in Materials in the online-only Data Supplement). Patients were preferably treated with angiotensin converting enzyme inhibitors or angiotensin receptor blockers and a calcium-channel blocker, except if these were contraindicated or if the patients already had controlled blood pressure with their current regimen. If the previously mentioned association was already in use and the systolic and diastolic blood pressure remained >130 mm Hg or 80 mm Hg, respectively, a combination with a thiazide diuretic was preferred. If a thiazide diuretic was contraindicated or if other medications were deemed necessary, then spironolactone or clonidine was used. Medications were reduced or discontinued if patients presented systolic blood pressure <110 mm Hg or diastolic blood pressure <70 mm Hg. For patients with systolic blood pressure between 110 and 130 mm Hg or diastolic blood pressure between 70 and 80 mm Hg associated with symptoms of orthostatic hypotension, dose reduction of antihypertensive medications was attempted. For patients submitted to bariatric surgery, the necessity of reintroducing antihypertensive medications was initially checked on a daily basis in the immediate postoperative period, in the first visit 1 week after the procedure, and in the remaining follow-up visits. Adherence to treatment was based on patient self-report.

Besides medical therapy, patients randomized to the gastric bypass group were submitted to Roux-en-Y gastric bypass performed by a single surgeon (Figure I in the online-only Data Supplement).

Patients from both groups received nutritional advice based on national statements for hypertension and obesity.^15^ A visit to a dietitian from the investigation team followed each medical visit at the hospital to reinforce the nutritional recommendations previously indicated. Nutritional advice in the medical therapy group was mainly directed at weight reduction and blood pressure control.^15–17^ Aimed at progressive weight loss over time, a total daily energy consumption calculated as 20 kcal/kg of ideal body weight per day was recommended among the patients. Similarly, for the improvement of blood pressure control, the ingestion of high-sodium food, such as snacks, sausages, and fast food, was discouraged, and the reduction of salt used for cooking at home or added to already prepared food was encouraged. Fruit and vegetable consumption was also recommended to increase potassium intake. For those patients submitted to Roux-en-Y gastric bypass, the nutritional advice included information about food consistency in the postoperative period. During nutritional visits, a detailed evaluation regarding diet tolerance was performed.

In addition, all patients received psychological and physical activity counseling and were treated for other comorbidities according to current guidelines.

### Data Collection and Assessment

At baseline, we collected data on demographic information, comorbidities, anthropometric values, use of medications, and laboratory values. We collected office blood pressure at baseline and months 1, 3, 6, and 12. Twenty-four-hour ambulatory blood pressure monitoring (ABPM) (the detailed method of 24-hour ABPM is described in Materials in the online-only Data Supplement),^18^ echocardiogram, ECG, and laboratory values were measured at baseline and 12 months.

### End Points

The primary outcome was a reduction of ≥30% of the total number of antihypertensive medications while maintaining office systolic and diastolic blood pressure <140 mm Hg and 90 mm Hg, respectively, at 12 months (eg, patients using 2 or 3 medications needed to reduce ≥1 medication to achieve the primary end point; patients using 4 or 5 medications need to reduce ≥2). Secondary end points included number of antihypertensive drugs, systolic and blood pressure (office and 24-hour ABPM), weight and BMI, waist circumference, fasting plasma glucose and glycohemoglobin, homeostasis model assessment of insulin resistance index, lipid profile (low-density lipoprotein cholesterol, high-density lipoprotein cholesterol, and triglycerides levels), uric acid, high-sensitivity C-reactive protein levels, interventricular septum diastolic thickness, a 10-year Framingham risk score, and adverse events.

We also measured secondary end points defined post hoc: remission of hypertension (defined as systolic and diastolic blood pressure <140 mm Hg and 90 mm Hg, respectively, without medications based on office blood pressure), remission of hypertension (defined as systolic and diastolic blood pressure <140 mm Hg and 90 mm Hg, respectively, without medications based on 24-hour ABPM), and reduction of ≥30% of the total number of antihypertensive medications while maintaining an office systolic blood pressure <120 mm Hg (SPRINT levels [Systolic Blood Pressure Intervention Trial])^19^ at 12 months.

### Statistical Analysis

The study was initially designed to enroll 60 patients. During trial conduct and while blinded to the study results, the executive committee decided to increase the sample size to 100 patients to improve statistical power. This revised sample provides 90% power to detect an increase in the probability of the primary end point from 10% in the medical therapy group to 40% in the gastric bypass group, assuming a 2-sided α of 5%.

Continuous variables with a normal distribution are reported as mean and standard deviation. Variables with a non-normal distribution are reported as medians and interquartile ranges. Categorical variables are summarized as frequencies. Main analysis followed the modified intention-to-treat principle, and missing values were imputed with a simple carry-over procedure if the patient had information of 6-month visit.^20^ We used the Fisher exact test to analyze the primary end point, and results are reported as rate ratios and 95% confidence intervals (CIs). Continuous end points were analyzed with adjustments for baseline values using repeated measure analysis of variance models. Variables that did not hold a normal distribution assumption were analyzed using generalized estimating equation models with distribution that better fit the data.

A post hoc analysis was performed to assess the proportion of patients with a reduction number of the total antihypertensive medications of ≥30% while keeping the SPRINT target.^19^ We conducted other 5 sensitivity analyses for the primary end point: complete-case analysis, per-protocol analysis, as-treated analysis, worst-case scenario, and multiple imputation analysis. Definitions are provided in Table II in the online-only Data Supplement. We also conducted an adjusted analysis for BMI, number of medications at baseline, 10-year Framingham risk score, basal insulin level at baseline, and duration of hypertension using *Poisson* regression model with robust variance. In all cases, results are presented as rate ratios and 95% CIs.

The significance level for the primary end point was 0.05. For all other end points, the significance level was 0.05 without adjustment for multiple comparisons. Because of this, all secondary end points and analyses should be interpreted as exploratory. Analyses were performed using R software, version 3.3.3 (R Foundation for Statistical Computing).

## Results

### Participant Characteristics

**Figure 1.**
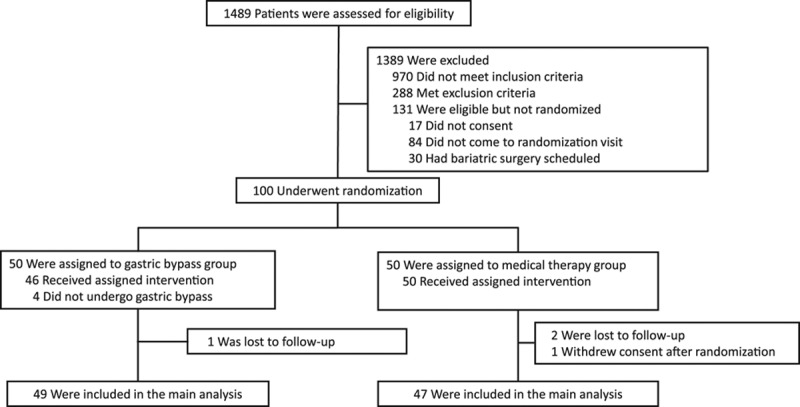
**Eligibility, randomization, and follow-up.**

Of the 100 included patients from May 2013 to May 2016, four patients were excluded from the final analysis. One patient withdrew consent after randomization in the medical therapy group, and 2 further patients in the medical therapy group and 1 in the gastric bypass group missed their follow-up visits. Thus, information on the primary end point at 12 months is available for 96 patients (Figure 1). Baseline characteristics are shown in Table 1. Groups were well balanced at baseline. The mean (±standard deviation) age was 43.8±9.2 years, 76% were women, the mean BMI was 36.9±2.7 kg/m2, and the average duration of hypertension was 9.4±6.7 years.

**Table 1.**
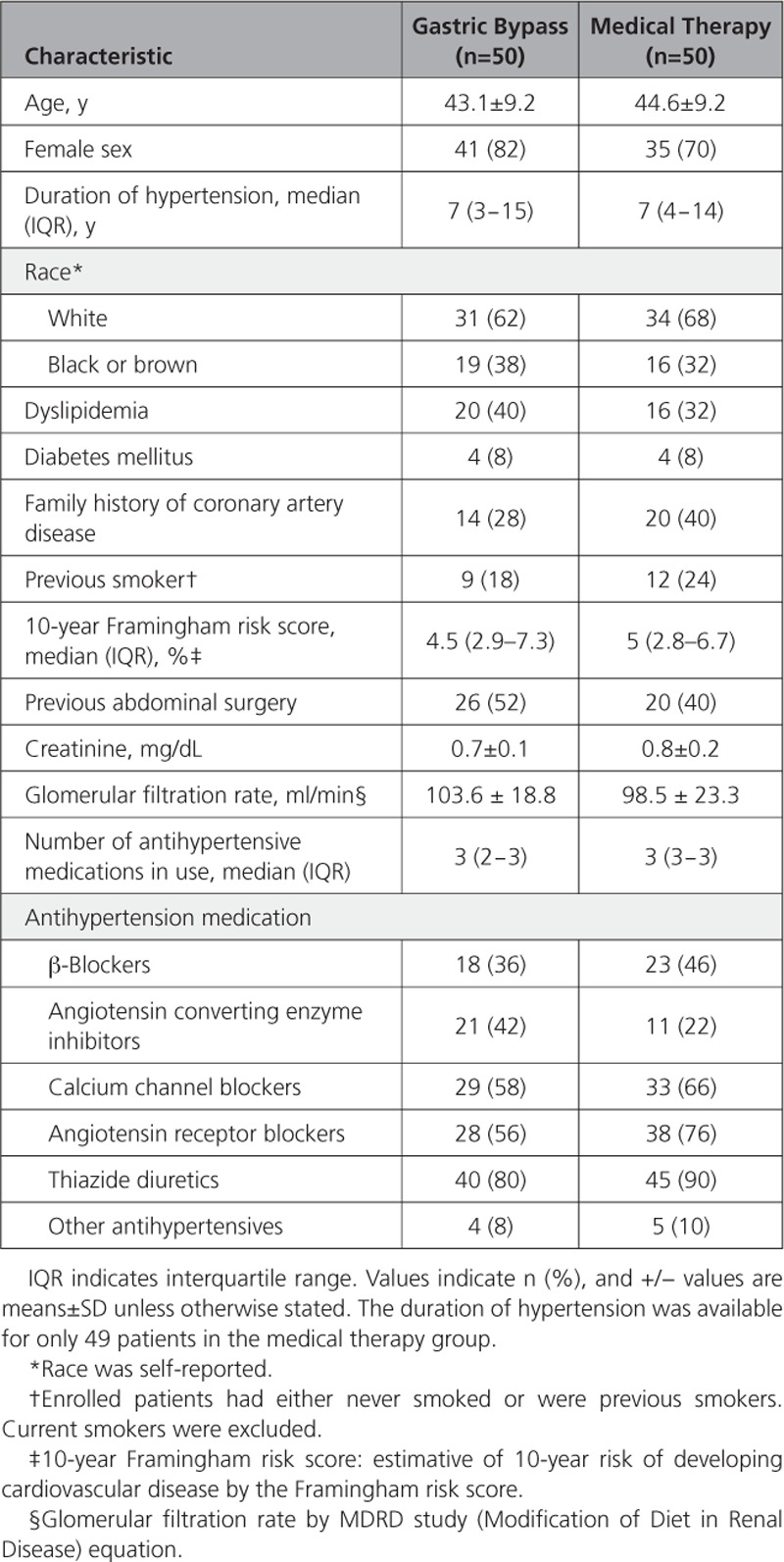
Baseline Characteristics of Study Participants

### Primary End Point and Effects on Blood Pressure

**Figure 2.**
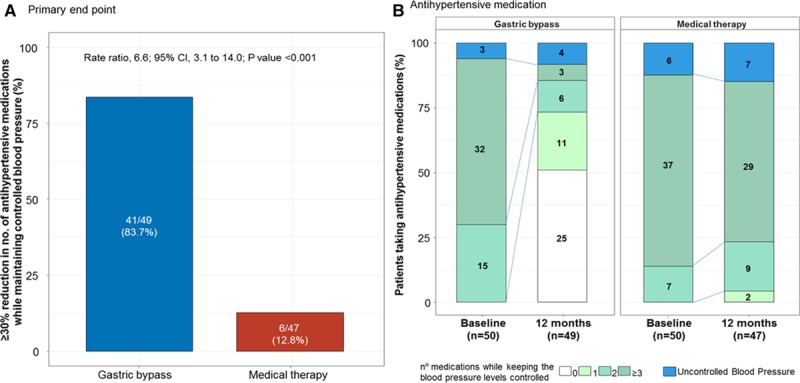
**Primary end point and medication use.** The proportion of patients with reduction of the total number of antihypertensive drugs of ≥30% while keeping the office blood pressures levels controlled. **A**, The *P* value for comparing proportions of patients with primary end point was performed using the Fisher exact test. **B**, The distribution of the number of antihypertensive medications used during the study period and patients with uncontrolled blood pressure (Office BP). CI indicates confidence interval.

Reduction of ≥30% of the total number of antihypertensive medications while maintaining controlled office blood pressure levels occurred in 41 of 49 patients from the gastric bypass group (83.7%) compared with 6 of 47 patients (12.8%) from the control group, with a rate ratio of 6.6 (95% CI, 3.1–14.0; *P*<0.001) (Figure 2A).

Twenty-five of 49 patients (51%) from the gastric bypass group showed remission of hypertension (defined as systolic and diastolic blood pressure <140 mm Hg and 90 mm Hg, respectively, without medications), whereas no patient submitted to medical therapy was free of antihypertensive drugs at 12 months (Figure 2B). Results were similar considering remission rates based on 24-hour ABPM. In this sense, 22 of 48 patients (45.8%) from the gastric bypass group showed remission of hypertension, whereas no patient submitted to medical therapy was free of antihypertensive drugs at 12 months. Additionally, a reduction in the number of antihypertensive medications (*P*<0.001) occurred, as well as in most classes of medications for blood pressure control (Table 2 and Table III in the online-only Data Supplement).

**Table 2.**
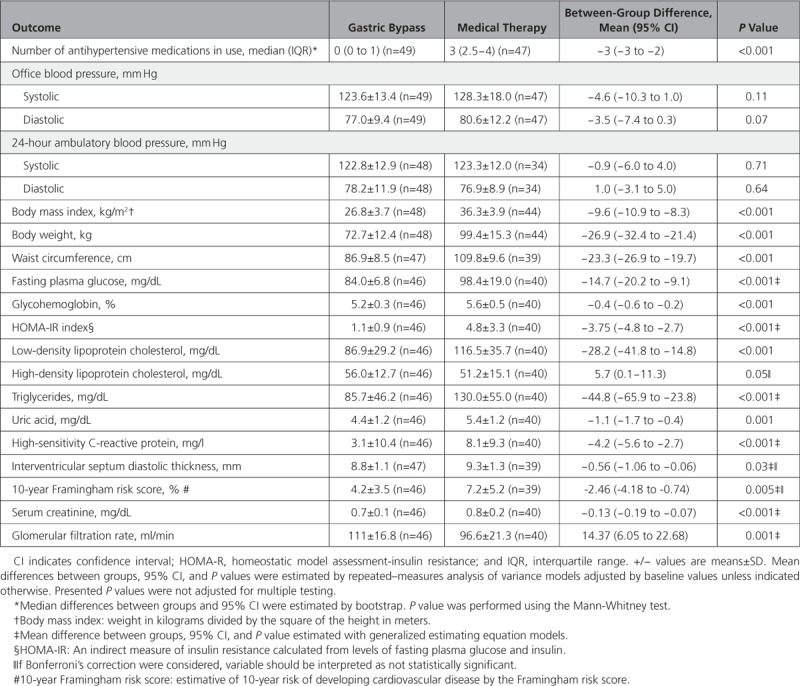
Secondary Outcomes at 12 Months

A post hoc analysis for the primary end point considering the SPRINT target reached consistent results with rate ratio of 3.8 (95% CI, 1.4–10.6; *P*=0.005). The reduction occurred in 16 of 49 patients from the gastric bypass group (32.7%) compared with 4 of 47 (8.5%) from the control group (Figure IIA in the online-only Data Supplement). Eleven patients (22.4%) from the gastric bypass group and none in the control group were able to achieve SPRINT levels without antihypertensives (Figure IIB in the online-only Data Supplement).

**Figure 3.**
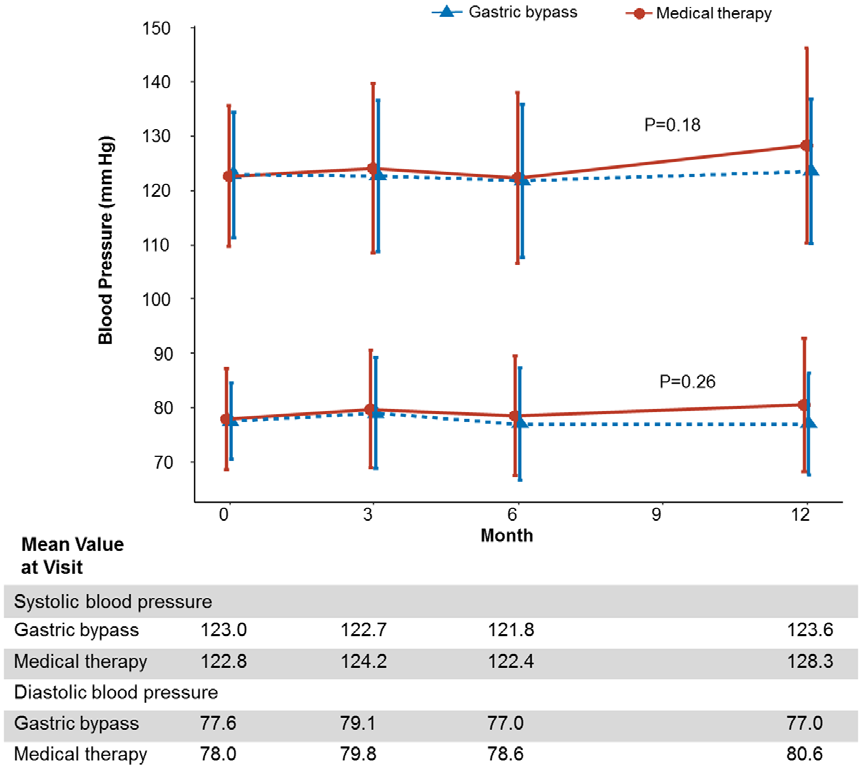
**Blood pressure.** The mean office blood pressure levels over a 12-month period among patients receiving medical therapy and those who underwent gastric bypass surgery. I bars indicate standard deviation. Mean values in each group are provided below the graphs.

Exploratory secondary end points are presented in Table 2 and Table IV in the online-only Data Supplement. The office blood pressure during the follow-up was similar in both groups (Table 2 and Figure 3). Blood pressure levels assessed by 24-hour ABPM were also similar between groups (Table V in the online-only Data Supplement).

### Weight Loss

**Figure 4.**
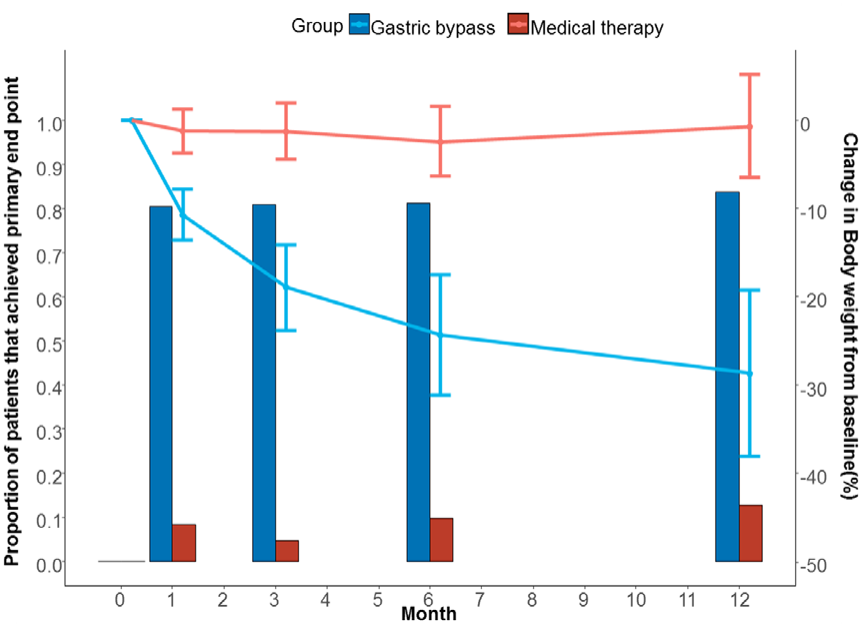
**Proportion of patients who achieved primary end point (bars graphs) and changes in body weight during 12 months (lines graphs).** Proportion of patients with reduction of the total antihypertensive drugs of ≥30% while maintaining the office blood pressure levels controlled (bar graph) and change of body weight (line graph). I bars indicate standard deviation.

At 12 months, changes in weight and BMI were greater in the gastric bypass than in the medical therapy group (Table 2 and Figure III in the online-only Data Supplement). The BMI was 26.8±3.7 kg/m^2^ in the surgical group and 36.3±3.9 kg/m^2^ in medical therapy group (*P*<0.001). Additionally, the waist circumference was lower in the gastric bypass group (86.9±8.5 cm) than in the control group (109.8.2±9.6 cm, *P*<0.001). Whereas weight decreased progressively during the 12-month follow-up in the gastric bypass group, the effect on the primary end point was fully achieved on the first month and was maintained during the 12-month follow-up period (Figure 4).

### Glycemic Control and Lipid Profile

Patients who underwent gastric bypass had lower levels of fasting plasma glucose (*P*<0.001), glycohemoglobin (*P*<0.001), and homeostasis model assessment of insulin resistance (*P*<0.001) (Table 2). The low-density lipoprotein cholesterol and triglycerides levels at 12 months were lower in the gastric bypass than in the medical therapy group (*P*<0.001 for both comparisons) (Table 2). Conversely, high-density lipoprotein cholesterol levels increased in the gastric bypass compared to the control group (*P*=0.05).

### Other End Points

A significant reduction in the high-sensitivity C-reactive protein level was found in the gastric bypass group compared with the medical therapy group (*P*<0.001). Uric acid was lower in the gastric bypass group than in the control group (*P*=0.001). Similarly, the interventricular septum diastolic thickness was lower in the gastric bypass group than in the control group (*P*=0.03), and the 10-year Framingham risk score was lower in the gastric bypass group than in the medical therapy group (*P*=0.005) (Table 2).

### Renal Function

Creatinine levels at 12 months were 0.7±0.1 mg/dL in the gastric bypass group and 0.8±0.2 mg/dL in the medical therapy group (<0.001). The glomerular filtration rate at 12 months was 111±16.8 mL/min in the gastric bypass group and 96.6±21.3 mL/min in the medical therapy group (*P*=0.001) (Table 2).

### Adverse Events

Table 3 shows adverse events at 12 months. Six patients needed hospitalization in the gastric bypass group versus none in the control group (*P*=0.03). Only 2 of them were hospitalized because of procedure complications. One patient required a reoperation 4 months after surgery because of an abscess near the jejunal anastomosis, and 1 was admitted for vomiting and dehydration. Four patients developed cholelithiasis after the gastric bypass, 3 were symptomatic, and 1 was asymptomatic. All of them were submitted to a laparoscopic cholecystectomy, and all recovered uneventfully. One patient developed an anastomotic ulcer and was successfully treated medically. Anemia was present in 11% and 10% of participants in the gastric bypass group and medical therapy group, respectively (*P*=1.00).

**Table 3.**
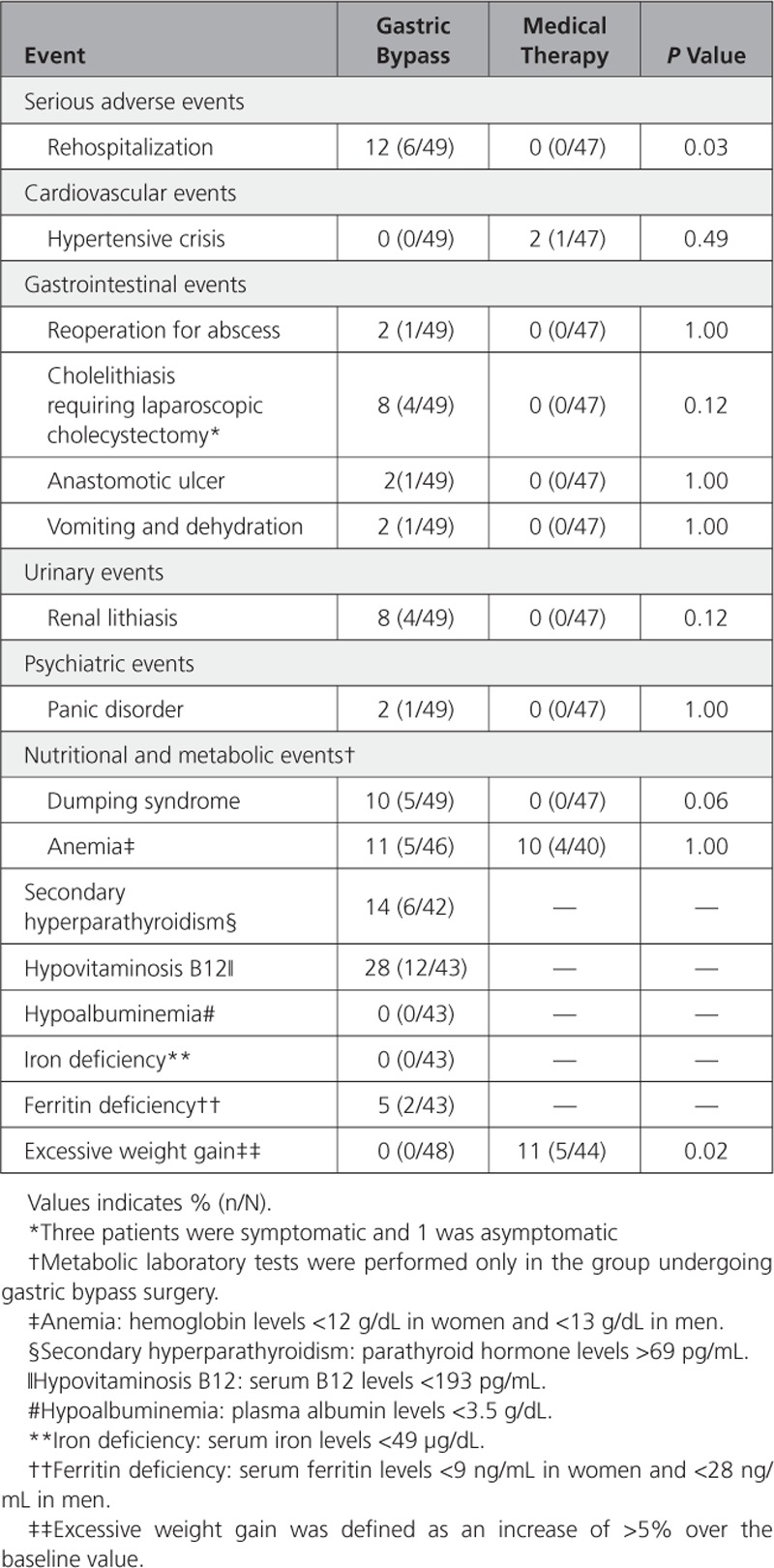
Adverse Events Through 12 Months

Nutritional parameters in the gastric bypass group showed significant differences from baseline to 12 months only for anemia, 0% to 11%, respectively (*P*=0.03), and hypovitaminosis B12, 9% to 28%, respectively (*P*=0.01). No difference for secondary hyperparathyroidism, hypoalbuminemia, iron, and ferritin deficiency occurred (Table VI in the online-only Data Supplement).

There were no deaths, episodes of severe hypoglycemia, malnutrition, or excessive weight loss. Five participants (11%) in the medical therapy group showed excessive weight gain (increase of >5% over the baseline value).

### Sensitivity Analyses

Sensitivity analyses are presented in Table II in the online-only Data Supplement. An analysis assessing treatment effect on the primary end point adjusted for BMI, number of medications at baseline, 10-year Framingham risk score, basal insulin level at baseline, and duration of hypertension found similar results with a rate ratio of 6.4 (95% CI, 3.1–13.0; *P*<0.001). Accordingly, results for the complete-case, per-protocol, as-treated, worst-case scenario, and multiple imputation analyses were consistent with those observed for the main analysis.

## Discussion

Our results indicated that at 12 months, patients with obesity and hypertension who underwent gastric bypass plus medical therapy were significantly more likely to reduce ≥30% of the number of medications while maintaining controlled blood pressure than patients managed with medical therapy alone. Notably, half of the patients in the surgical group were able to maintain systolic and diastolic blood pressure <140 mm Hg and 90 mm Hg, respectively, without the need for medications (remission of hypertension), whereas no control group patient was free of medications at 12 months. The post hoc analysis indicated that ≈20% of patients in the gastric bypass group achieved SPRINT goals^19^ without medications at 12 months. In addition, exploratory end points such as the number of antihypertensive medications, waist circumference, BMI, fasting plasma glucose, insulin resistance, glycohemoglobin, low-density lipoprotein cholesterol, triglycerides, high-sensitivity C-reactive protein, uric acid, and the 10-year Framingham risk score were lower in the surgical than in the medical therapy group. Thus, despite the fact that the groups had similar blood pressure levels at 12 months, patients submitted to gastric bypass were able to achieve these levels with few or any medications and also had their metabolic profile improved.

Several observational studies suggested improvement and remission of hypertension after surgery.^6,7,9,21^ Previous randomized trials in patients with diabetes mellitus also assessed the effects of bariatric surgery on hypertension control. In the STAMPEDE trial (Surgical Treatment and Medications Potentially Eradicate Diabetes Efficiently Trial), the authors observed a significant reduction in the number of antihypertensives after surgery at 12 months, and ≈60% of the patients were able to stop their medications while maintaining hypertension control.^8^ After 5 years, the number of patients using >3 cardiovascular drugs fell from 61.2% in the baseline to 20.4%.^22^ In the study by Mingrone et al,^10^ antihypertensive therapy was reduced or discontinued in 80% of the patients undergoing gastric bypass. Other trials also documented improvement of hypertension after bariatric surgery.^23,24^

Our trial confirms the results from the observational studies with better control of residual confounding because of the randomized design. In contrast with the GATEWAY trial, in previous randomized trials, hypertension improvement and remission were measured as secondary end points. In addition, hypertension management and decision to reduce or discontinue antihypertensive medications were not standardized in all studies. Finally, the GATEWAY trial included a broader population of patients with obesity and hypertension, the majority of whom did not have diabetes mellitus. Therefore, our trial provides novel findings and complements the results from the available randomized evidence.

Although hypertension improvement after bariatric surgery could be attributable to hemodynamic changes and decreased intra-abdominal pressure associated with weight loss, it is likely that several factors play an important role.^25,26^ Insulin resistance is associated with renal sodium reabsorption and increased sympathetic tone.^27^ Another factor that may influence hypertension control is inflammation, which can modulate arterial stiffness.^28^ Small mechanistic studies suggest that this modulation is attributable to a reduction in perivascular adipocyte inflammation. In lean healthy subjects, the perivascular adipose tissue exerts an anticontratile effect on adjacent small arteries, which is lost in patients with obesity probably because of inflammation.^29^ Thus, by reversing inflammation, bariatric surgery can contribute to the restoration of normal anticontratile activity. We demonstrated both a significant reduction in homeostasis model assessment of insulin resistance index and high-sensitivity CRP levels in the gastric bypass group when compared with controls. Reduction of such factors is not immediate after surgery. In our trial, hypertension improvement occurred early after surgery and was maintained for ≤12 months (which is consistent with previous studies) (Figure 4).^30^ Thus, the effects could also be attributable to additional mechanisms.^31^ Some evidence has shown that gut hormones may be involved in sodium and water handling of the kidney.^31^ In this sense, glucagon-like peptide-1 and peptide YY, whose effects are exaggerated within days after surgery, may act as mediators between the gut and the kidney (enterorenal axis concept), influencing electrolyte transport in the renal tubular cells as well as causing diuresis.^32^

Our study has limitations that merit consideration. These limitations include the relatively short duration of follow-up (12 months), being a single-center trial, and the open-label nature of the study (which increased the risk of bias because doctors could have preferentially moderated therapy for subjects they knew had undergone gastric bypass). We attempted to minimize the potential systematic errors associated with the lack of masking of investigators by training all the personnel involved in the study and guaranteeing that they strictly follow the trial protocol. A single surgeon with long-term expertise in bariatric surgery performed all the surgeries. Thus, one may question the external validity of our findings, especially to sites with less experience in these procedures. Because of the study’s academic nature (investigator-initiated) and the consequent limited funding, in our trial, compliance with study medications was based on patient self-report. Therefore, the risk of verification bias in our primary end point represents a major limitation of our trial. At 12 months, information on the primary end point was not available for 4 out of 100 patients. Nevertheless, our findings for the primary end point were robust to all sensitivity analyses assumptions. We excluded patients with systolic blood pressure ≥180 mm Hg and diastolic blood pressure ≥120  Hg as well as elderly patients. Whether our results can be extrapolated to these populations remains to be determined. Finally, we are not able to generalize from a study cohort containing patients with class I obesity (BMI 30–34.9) to the overwhelming number of individuals who undergo bariatric surgery, those with class II and III obesity (BMI >35).

In conclusion, bariatric surgery represents an effective strategy for reducing antihypertensive drugs at 12 months in patients with obesity and hypertension while maintaining controlled blood pressure levels. The durability of our findings remains uncertain, but further 4-year follow-up of patients should allow assessment of the long-term effects of bariatric surgery in the studied population. Given the morbidity of surgery, our results do not imply that all patients with obesity and hypertension with similar characteristics to those included in our trial should be submitted to bariatric surgery. However, multiple barriers exist to managing hypertension in obesity, including nonadherence to long-term multiple antihypertensive drugs. Thus, gastric bypass represents 1 extra option to help achieve blood pressure control with the added benefit of improving metabolic and inflammatory profile. Taken together, such effects have the potential to reduce major cardiovascular events, although further trials are needed to confirm these benefits.

## Acknowledgments

The authors thank the Surgical Center and Ward staff and the Surgical Team and Research Institute-Heart Hospital for assistance with the GATEWAY trial.

## Disclosures

Dr Schiavon received a significant research grant from, participated in a Speakers Bureau for, and received honoraria from Johnson & Johnson Brazil. Dr Cohen received a significant research grant from Johnson & Johnson Brazil; participated in a Speakers Bureau for Johnson & Johnson Brazil; and served as a significant consultant on the advisory board of GI Dynamics. Dr Gonçalves de Souza participated in a Speakers Bureau for Daiichi-Sankyo Brasil Farmacêutica and served as a modest consultant on the advisory board of Aché Laboratório Farmacêutico. Dr Drager received a research grant from Philips Respironics and participated in a Speakers Bureau for Johnson & Johnson Brazil. Dr Berwanger received a significant research grant from AstraZeneca, Boheringer Ingelheim, Sanofi, Amgen, Pfizer, Roche, and Bayer. The other authors have nothing to disclose.

## Supplementary Material

## References

[1] The GBD 2015 Obesity Collaborators (2017). Health effects of overweight and obesity in 195 countries over 25 years.. N Engl J Med.

[2] Forouzanfar MH, Liu P, Roth GA, Ng M, Biryukov S, Marczak L, Alexander L, Estep K, Hassen Abate K, Akinyemiju TF, Ali R, Alvis-Guzman N, Azzopardi P, Banerjee A, Bärnighausen T, Basu A, Bekele T, Bennett DA, Biadgilign S, Catalá-López F, Feigin VL, Fernandes JC, Fischer F, Gebru AA, Gona P, Gupta R, Hankey GJ, Jonas JB, Judd SE, Khang YH, Khosravi A, Kim YJ, Kimokoti RW, Kokubo Y, Kolte D, Lopez A, Lotufo PA, Malekzadeh R, Melaku YA, Mensah GA, Misganaw A, Mokdad AH, Moran AE, Nawaz H, Neal B, Ngalesoni FN, Ohkubo T, Pourmalek F, Rafay A, Rai RK, Rojas-Rueda D, Sampson UK, Santos IS, Sawhney M, Schutte AE, Sepanlou SG, Shifa GT, Shiue I, Tedla BA, Thrift AG, Tonelli M, Truelsen T, Tsilimparis N, Ukwaja KN, Uthman OA, Vasankari T, Venketasubramanian N, Vlassov VV, Vos T, Westerman R, Yan LL, Yano Y, Yonemoto N, Zaki ME, Murray CJ (2017). Global burden of hypertension and systolic blood pressure of at least 110 to 115 mm Hg, 1990–2015.. JAMA.

[3] Gu Q, Burt VL, Dillon CF, Yoon S (2012). Trends in antihypertensive medication use and blood pressure control among United States adults with hypertension: the National Health and Nutrition Examination Survey, 2001 to 2010.. Circulation.

[4] Reisin E, Graves JW, Yamal JM, Barzilay JI, Pressel SL, Einhorn PT, Dart RA, Retta TM, Saklayen MG, Davis BR, ALLHAT Collaborative Research Group (2014). Blood pressure control and cardiovascular outcomes in normal-weight, overweight, and obese hypertensive patients treated with three different antihypertensives in ALLHAT.. J Hypertens.

[5] Arterburn DE, Olsen MK, Smith VA, Livingston EH, Van Scoyoc L, Yancy WS, Eid G, Weidenbacher H, Maciejewski ML (2015). Association between bariatric surgery and long-term survival.. JAMA.

[6] Adams TD, Davidson LE, Litwin SE, Kim J, Kolotkin RL, Nanjee MN, Gutierrez JM, Frogley SJ, Ibele AR, Brinton EA, Hopkins PN, McKinlay R, Simper SC, Hunt SC (2017). Weight and metabolic outcomes 12 years after gastric bypass.. N Engl J Med.

[7] Hallersund P, Sjöström L, Olbers T, Lönroth H, Jacobson P, Wallenius V, Näslund I, Carlsson LM, Fändriks L (2012). Gastric bypass surgery is followed by lowered blood pressure and increased diuresis: long term results from the Swedish Obese Subjects (SOS) study.. PLoS One.

[8] Schauer PR, Kashyap SR, Wolski K, Brethauer SA, Kirwan JP, Pothier CE, Thomas S, Abood B, Nissen SE, Bhatt DL (2012). Bariatric surgery versus intensive medical therapy in obese patients with diabetes.. N Engl J Med.

[9] Wilhelm SM, Young J, Kale-Pradhan PB (2014). Effect of bariatric surgery on hypertension: a meta-analysis.. Ann Pharmacother.

[10] Mingrone G, Panunzi S, De Gaetano A, Guidone C, Iaconelli A, Leccesi L, Nanni G, Pomp A, Castagneto M, Ghirlanda G, Rubino F (2012). Bariatric surgery versus conventional medical therapy for type 2 diabetes.. N Engl J Med.

[11] Research Institute-HCor-INSTITUTIONAL DATA REPOSITORY (2017). BR São Paulo, São Paulo.. http://www.hcor.com.br/en/.

[12] Schiavon CA, Ikeoka DT, de Sousa MG, Silva CR, Bersch-Ferreira AC, de Oliveira JD, Noujaim PM, Cohen RV, Amodeo C, Berwanger O, GATEWAY (GAstric bypass surgery to TrEat patients With steAdy hYpertension) Investigators (2014). Effects of gastric bypass surgery in patients with hypertension: rationale and design for a randomised controlled trial (GATEWAY study).. BMJ Open.

[13] James PA, Oparil S, Carter BL, Cushman WC, Dennison-Himmelfarb C, Handler J, Lackland DT, LeFevre ML, MacKenzie TD, Ogedegbe O, Smith SC, Svetkey LP, Taler SJ, Townsend RR, Wright JT, Narva AS, Ortiz E (2014). 2014 evidence-based guideline for the management of high blood pressure in adults: report from the panel members appointed to the Eighth Joint National Committee (JNC 8).. JAMA.

[14] Law MR, Wald NJ, Morris JK, Jordan RE (2003). Value of low dose combination treatment with blood pressure lowering drugs: analysis of 354 randomised trials.. BMJ.

[15] (2010). Sociedade Brasileira de Cardiologia, Sociedade Brasileira de Hipertensão, Sociedade Brasileira de Nefrologia.. Arg Bras Cardiol.

[16] ABESO (2009). Diretrizes Brasileiras de Obesidade.. In: ABESO.

[17] Chobanian AV, Bakris GL, Black HR, Cushman WC, Green LA, Izzo JL, Jones DW, Materson BJ, Oparil S, Wright JT, Roccella EJ, National Heart, Lung, and Blood Institute Joint National Committee on Prevention, Detection, Evaluation, and Treatment of High Blood Pressure; National High Blood Pressure Education Program Coordinating Committee (2003). The seventh report of the Joint National Committee on Prevention, Detection, Evaluation, and Treatment of High Blood Pressure: the JNC 7 report.. JAMA.

[18] Sociedade Brasileira de Cardiologia, Sociedade Brasileira de Hipertensão (2011). V Diretrizes de Monitorização Ambulatorial da Pressão Arterial (MAPA) e III Diretrizes de Monitorização Residencial da Pressão Arterial (MRPA)..

[19] The SPRINT Research Group (2015). A randomized trial of intensive versus standard blood-pressure control.. N Engl J Med.

[20] Shao J, Zhong B (2003). Last observation carry-forward and last observation analysis.. Stat Med.

[21] Vest AR, Heneghan HM, Agarwal S, Schauer PR, Young JB (2012). Bariatric surgery and cardiovascular outcomes: a systematic review.. Heart.

[22] Schauer PR, Bhatt DL, Kirwan JP, Wolski K, Aminian A, Brethauer SA, Navaneethan SD, Singh RP, Pothier CE, Nissen SE, Kashyap SR, STAMPEDE Investigators (2017). Bariatric surgery versus intensive medical therapy for diabetes: 5-year outcomes.. N Engl J Med.

[23] Ikramuddin S, Billington CJ, Lee WJ, Bantle JP, Thomas AJ, Connett JE, Leslie DB, Inabnet WB, Jeffery RW, Chong K, Chuang LM, Sarr MG, Jensen MD, Vella A, Ahmed L, Belani K, Schone JL, Olofson AE, Bainbridge HA, Laqua PS, Wang Q, Korner J (2015). Roux-en-Y gastric bypass for diabetes (the Diabetes Surgery Study): 2-year outcomes of a 5-year, randomised, controlled trial.. Lancet Diabetes Endocrinol.

[24] Liang Z, Wu Q, Chen B, Yu P, Zhao H, Ouyang X (2013). Effect of laparoscopic Roux-en-Y gastric bypass surgery on type 2 diabetes mellitus with hypertension: a randomized controlled trial.. Diabetes Res Clin Pract.

[25] Auclair A, Biertho L, Marceau S, Hould F, Biron S, Lebel S, Julien F, Lescelleur O, Lacasse Y, Piché M, Cianflone K, Parlee SD, Goralski K, Martin J, Bastien M, St-pierre DH, Poirier P Bariatric surgery-induced resolution of hypertension and obstructive sleep apnea: impact of modulation of body fat, ectopic fat, autonomic nervous activity, inflammatory and adipokine profiles.. Obes Surg.

[26] Schiavon CA, Drager LF, Bortolotto LA, Amodeo C, Ikeoka D, Berwanger O, Cohen RV (2016). The role of metabolic surgery on blood pressure control.. Curr Atheroscler Rep.

[27] Bueter M, Ahmed A, Ashrafian H, le Roux CW (2009). Bariatric surgery and hypertension.. Surg Obes Relat Dis.

[28] Arner P, Bäckdahl J, Hemmingsson P, Stenvinkel P, Eriksson-Hogling D, Näslund E, Thorell A, Andersson DP, Caidahl K, Rydén M (2015). Regional variations in the relationship between arterial stiffness and adipocyte volume or number in obese subjects.. Int J Obes (Lond).

[29] Aghamohammadzadeh R, Greenstein AS, Yadav R, Jeziorska M, Hama S, Soltani F, Pemberton PW, Ammori B, Malik RA, Soran H, Heagerty AM (2013). Effects of bariatric surgery on human small artery function: evidence for reduction in perivascular adipocyte inflammation, and the restoration of normal anticontractile activity despite persistent obesity.. J Am Coll Cardiol.

[30] Ahmed AR, Rickards G, Coniglio D, Xia Y, Johnson J, Boss T, O’Malley W (2009). Laparoscopic Roux-en-Y gastric bypass and its early effect on blood pressure.. Obes Surg.

[31] le Roux CW, Welbourn R, Werling M, Osborne A, Kokkinos A, Laurenius A, Lönroth H, Fändriks L, Ghatei MA, Bloom SR, Olbers T (2007). Gut hormones as mediators of appetite and weight loss after Roux-en-Y gastric bypass.. Ann Surg.

[32] Michell AR, Debnam ES, Unwin RJ (2008). Regulation of renal function by the gastrointestinal tract: potential role of gut-derived peptides and hormones.. Annu Rev Physiol.

