# Supplementary material for: Effects of Bariatric Surgery in Obese Patients With Hypertension: The GATEWAY Randomized Trial (Gastric Bypass to Treat Obese Patients With Steady Hypertension)
Source: Circulation. 2019 Oct 8;137(11):1132–42. doi: 10.1161/CIRCULATIONAHA.117.032130 (PMC5865494; doi:10.1161/CIRCULATIONAHA.117.032130)
Supplement: Supplementary file 1 [file cir-137-1132-s001.pdf]

SUPPLEMENTAL MATERIAL

Effects of Bariatric Surgery in Obese Patients With Hypertension: The GATEWAY Randomized Trial

Carlos Aurelio Schiavon,[1] Angela Cristine Bersch-Ferreira,[1] Eliana Vieira Santucci,[1] Juliana Dantas Oliveira,[1] Camila Ragne Torreglosa,[1] Priscila Torres Bueno,[1] Julia Caldas Frayha,[1] Renato Nakagawa Santos,[1] Lucas Petri Damiani,[1] Patricia Malvina Noujaim,[2] Helio Halpern,[2] Frederico L. J. Monteiro,[2] Ricardo Vitor Cohen,[3] Carlos H. Uchoa,[4] Marcio Gonçalves de Souza,[5] Celso Amodeo,[5] Luiz Bortolotto,[4] Dimas Ikeoka,[6] Luciano Ferreira Drager,[4] Alexandre Biasi Cavalcanti,[1] Otavio Berwanger[1]

[1]Research Institute - Heart Hospital (HCor) - São Paulo, Brazil.
[2]Surgical Center - Heart Hospital (HCor) - São Paulo, Brazil.
[3]Oswaldo Cruz German Hospital - São Paulo, Brazil.
[4]Heart Institute (InCor), Hypertension Unit - São Paulo, Brazil.
[5]Dante Pazzanese Institute of Cardiology – Department of Hypertension - São Paulo, Brazil.
[6]Intensive Unit - Heart Hospital (HCor) - São Paulo, Brazil.

Table of Contents

Item	Description	Page
Supplemental Methods	Box 1. Criteria for eligibility	3
Supplemental Methods	Detailed methods of blood pressure measurement	4
Supplemental Table 1	Definitions of dose ranges of antihypertensive medications	5
Supplemental Table 2	Medications	7
Supplemental Table 3	Secondary end points.	9
Supplemental Table 4	24-hour ambulatory blood pressure monitoring at 12 months	13
Supplemental Table 5	Nutritional parameters	14
Supplemental Table 6	Sensitivity analyses of gastric bypass versus medical therapy effect on the primary end point	15
Supplemental Figure 1	Roux-en-Y gastric bypass	17
Supplemental Figure 2	Proportion of patients with reduction of the total number of antihypertensive drugs at least 30% while maintaining systolic blood pressure lower than 120 mm Hg (SPRINT target), Antihypertensive medication distribution.	18

Supplemental Figure 3	Body-mass index	19
Supplemental figure titles and legends	Supplemental Figures 1, 2 and 3	20
References	References	21

Supplemental Methods

Box 1. Criteria for eligibility

**Inclusion Criteria**
Age: 18 to 65 years<br><br>Body mass index (BMI) in the range from 30.0 to 39.9<br><br>Previously diagnosed arterial hypertension (as defined by current use of at least two full-dose anti-hypertensive drugs or more than two in moderate doses)
**Exclusion Criteria**
Severe and uncontrolled arterial hypertension (≥ 180/120 mmHg)<br><br>Cerebrovascular disease with acute events or alteration in the cognitive function in the past six months<br><br>Heart diseases (myocardial infarction, angina, coronary revascularization, heart failure) occurred or diagnosed in the past 6 months<br><br>Severe psychiatric disorders: schizophrenia, bipolar disorder, severe depression, psychosis<br><br>Severe kidney disease: diabetic nephropathy, important reduction of renal function (glomerular filtration rate < 30 ml/min)<br><br>Diagnosed secondary hypertension, except due to sleep apnea<br><br>Advanced peripheral arterial disease<br><br>Type I (any) or uncontrolled type II (with HbA1c>7,0) diabetes mellitus or latent autoimmune diabetes of adults (LADA)<br><br>Atrophic gastritis<br><br>Alcoholism or use of illicit drugs<br><br>Current tobacco smoking habit<br><br>Previous abdominal surgery (except for MacBurney, Pfanneistil and laparoscopic cholecistectomy)<br><br>Severe hepatic diseases<br><br>Pregnancy or women at childbearing risk who are not using effective contraceptive methods<br><br>Cancer in the last five years<br><br>Current use of immunosuppressive drugs, chemotherapy or radiotherapy<br><br>Inability to understand and adhere to the treatment or post-surgical instructions

Detailed methods of blood pressure measurement

Detailed method of office blood pressure measurement

All office blood pressure measurements were taken with a calibrated aneroid sphygmomanometer (Durashock DS44 TYCOS - Welch Allyn®, which is deemed to be accurate by the AAMI). Three seated blood pressure measurements were obtained, at least one minute apart between measurements, after resting for five minutes. We used an appropriate cuff size adjusted to the circumference of the arm. Patients were requested to take all antihypertensive medications at least one hour prior to the blood pressure measurements, and were instructed not to drink coffee, or alcohol, smoke or exercise within 120 minutes before measurements.

Detailed method of 24-hour ambulatory blood pressure measurement (ABPM)

Twenty-four-hour ABPM was evaluated using the Dyna-mapa – CARDIOS device, which is deemed to be accurate by the AAMI and BHS (British Hypertension Society). The ABPM parameters were set for every 15 minutes throughout the day (7 am to 10:59 pm) and for every 30 minutes at night (11 pm to 6:59 am). Patients were asked to keep a diary of key activities (going to bed and getting up, taking medications, other significant events). A 24-hour ABPM was considered adequate if the number of successful daytime readings captured was ≥16 and the number of successful nighttime readings captured was ≥8. Patients were classified as having normal 24-hour BP if the corresponding values were SBP<125 mm Hg and DBP<75 mm Hg. The normal awake BP corresponded to the values of SBP<130 mm Hg and DBP<85 mm Hg. The normal sleep BP corresponded to the values of SBP<110 mm Hg and DBP<70 mm Hg. Twenty-four-hour ABPM was done according to The V Brazilian Guidelines for Ambulatory Blood Pressure Monitoring (ABPM).[1]

Supplemental Table 1. Definitions of dose ranges of antihypertensive medications

	**Dose Range (mg)**
**Angiotensin converting enzyme inhibitor**	
Enalapril	5 to 40
Captopril	12.5 to 50
Ramipril	2.5 to 10
Lisinopril	5 to 40
Perindopril	5 to 10
**Angiotensin receptor blockers**	
Candesartan	8 to 32
Losartan	25 to 100
Olmesartan	20 to 40
Telmisartan	40 to 80
Valsartan	80 to 320
**Calcium channel blockers**	
Amlodipine	2.5 to 10
Lercanidipine	10 to 20
Diltiazem	90 to 360
Levamlodipine	2.5 to 5
**Diuretics**	
**Thiazide**	
Chlorthalidone	12.5 to 50
Hydroclhorothiazide	12.5 to 50
Indapamide	1.5 to 5
**Potassium-sparing**	
Spironolactone	12.5 to 100
Amiloride	2.5 to 5

	Dose Range (mg)
**Beta-blockers**	
Atenol	25 to 100
Metoprolol	25 to 200
Propranolol	40 to 320

Supplemental Table 2. Medications

	Baseline		Month 3		Month 6		Month 12		
	**Gastric Bypass**	**Medical Therapy**	**Gastric Bypass**	**Medical Therapy**	**Gastric Bypass**	**Medical Therapy**	**Gastric Bypass**	**Medical Therapy**	**P Value**
Number of antihypertensive drugs in use									**<0.001**
0	0 (0/50)	0 (0/50)	43 (20/47)	0 (0/42)	46 (22/48)	0 (0/41)	53 (26/49)	0 (0/47)	
1	0 (0/50)	0 (0/50)	45 (21/47)	0 (0/42)	40 (19/48)	2 (1/41)	29 (14/49)	4 (2/47)	
2	30 (15/50)	14 (7/50)	6 (3/47)	17 (7/42)	6 (3/48)	17 (7/41)	12 (6/49)	21 (10/47)	
3	62 (31/50)	62 (31/50)	6 (3/47)	55 (23/42)	6 (3/48)	54 (22/41)	4 (2/49)	45 (21/47)	
4	6 (3/50)	20 (10/50)	0 (0/47)	26 (11/42)	2 (1/48)	24 (10/41)	2 (1/49)	25 (12/47)	
5	2 (1/50)	4 (2/50)	0 (0/47)	2 (1/42)	0 (0/48)	2 (1/41)	0 (0/49)	4 (2/47)	
Antihypertensive drugs in use	2.8±0.6 (n=50)	3.1±0.7 (n=50)	0.8±0.8 (n=47)	3.1±0.7 (n=42)	0.8±1.0 (n=48)	3.1±0.8 (n=41)	0.7±1.0 (n=49)	3±0.9 (n=47)	
Beta-Blockers	36 (18/50)	46 (23/50)	19 (9/47)	45 (19/42)	19 (9/48)	46 (19/41)	12 (6/49)*	43 (20/47)	**0.001**
Angiotensin converting enzyme inhibitors	42 (21/50)	22 (11/50)	21 (10/47)	17 (7/42)	21 (10/48)	22 (9/41)	16 (8/49)†	19 (9/47)	0.79
Calcium channel blockers	58 (29/50)	66 (33/50)	2 (1/47)	67 (28/42)	6 (3/48)	63 (26/41)	6 (3/49)†	64 (30/47)	**<0.001**
Angiotensin receptor blockers	56 (28/50)	76 (38/50)	28 (13/47)	83 (35/42)	23 (11/48)	78 (32/41)	26 (13/49)†	81 (38/47)	**<0.001**
Diuretics	80 (40/50)	92 (46/50)	6 (3/47)	90 (38/42)	8 (4/48)	83 (34/41)	10 (5/49)†	78 (37/47)	**<0.001**
Thiazide diuretics	100 (40/40)	98 (45/46)	100 (3/3)	100 (38/38)	75 (3/4)	100 (34/34)	80 (4/5)	97 (36/37)	0.23

	Baseline		Month 3		Month 6		Month 12		
	**Gastric Bypass**	**Medical Therapy**	**Gastric Bypass**	**Medical Therapy**	**Gastric Bypass**	**Medical Therapy**	**Gastric Bypass**	**Medical Therapy**	**P Value**
Other antihypertensives	8 (4/50)	10 (5/50)	0 (0/47)	9 (4/42)	2 (1/48)	10 (4/41)	2 (1/49)	13 (6/47)	0.06

Values are % (n/N). All P values in the footnotes were calculated on the basis of the 12-month data with the medical therapy groups as the comparator.

* P value = 0.006 for comparing baseline and 12-months was performed using the McNemar Test

† P value < 0.001 comparing baseline and 12-months was performed using the McNemar Test

Supplemental Table 3. Secondary End Points.

**End point**	**Gastric bypass**	**Medical therapy**	**P value**
Systolic blood pressure – office – mm Hg			
Baseline	123.0±11.6 (n=50)	122.8±12.9 (n=50)	
1 year	123.6±13.4 (n=49)	128.3±18.0 (n=47)	
Change from baseline	0.8±15.3 (n=49)	5.2±20.2 (n=47)	0.18
Diastolic blood pressure – office – mm Hg			
Baseline	77.6±7.0 (n=50)	78.0±9.3 (n=50)	
1 year	77.0±9.4 (n=49)	80.6±12.2 (n=47)	
Change from baseline	-0.4±12.4 (n=49)	2.4±15.2 (n=47)	0.26
Systolic blood pressure – 24-hour ABPM – mm Hg			
Baseline	118.8±9.2 (n=49)	122.5±12.2 (n=48)	
1 year	122.8±12.9 (n=48)	123.3±12.0 (n=34)	
Change from baseline	4.0±13.3 (n=47)	1.5±11.8 (n=33)	0.29
Diastolic blood pressure – 24-hour ABPM – mm Hg			
Baseline	73.6±6.8 (n=49)	76.5±9.4 (n=48)	
1 year	78.2±11.9 (n=48)	76.9±8.9 (n=34)	
Change from baseline	4.6±11.5 (n=47)	0.8±7.2 (n=33)	0.07
Body-mass index - kg/m2 *			
Baseline	37.4±2.4 (n=50)	36.4±2.9 (n=50)	
1 year	26.8±3.7 (n=48)	36.3±3.9 (n=44)	
Change from baseline	-10.8±3.7 (n=48)	-0.2±2.2 (n=44)	**<0.001**
Body weight - kg			
Baseline	102 ± 13.6 (n=50)	100.1 ± 14 (n=50)	
1 year	72.7 ± 12.4 (n=48)	99.4 ± 15.3 (n=44)	
Change from baseline	-29.5 ± 11.2 (n=48)	-0.7 ± 6 (n=44)	**<0.001**

End point	Gastric bypass	Medical therapy	P value
Waist circumference - cm			
Baseline	112.2±7.9 (n=50)	111±8.8 (n=48)	
1 year	86.9±8.5 (n=47)	109.8±9.6 (n=39)	
Change from baseline	-25.7±9.6 (n=47)	-0.9±6.3 (n=38)	**<0.001**
Glycated hemoglobin - %			
Baseline	5.7±0.5 (n=50)	5.7±0.7 (n=50)	
1 year	5.2±0.3 (n=46)	5.6±0.5 (n=40)	
Change from baseline	-0.48±0.38 (n=46)	-0.14±0.42 (n=40)	**<0.001**
Fasting plasma glucose - mg/dl			
Baseline	99.8±17.8 (n=50)	99.7±20.1 (n=50)	
1 year	84.0±6.8 (n=46)	98.4±19.0 (n=40)	
Change from baseline	-16.5±15.9 (n=46)	-1.6±9.9 (n=40)	**<0.001†**
HOMA-IR index - units‡			
Baseline	4.7 ± 2.6 (n=50)	5 ± 2.7 (n=50)	
1 year	1.1 ± 0.9 (n=46)	4.8 ± 3.3 (n=40)	
Change from baseline	-3.6 ± 2.5 (n=46)	-0.1 ± 3.4 (n=40)	**<0.001†**
Low-density lipoprotein cholesterol - mg/dl			
Baseline	121.5±30.4 (n=50)	120.8±34.9 (n=50)	
1 year	86.9±29.2 (n=46)	116.5±35.7 (n=40)	
Change from baseline	-35.0±30.2 (n=46)	-7.4±34.7 (n=40)	**<0.001**
High-density lipoprotein cholesterol - mg/dl			
Baseline	46.4±14.8 (n=50)	48.3±12.8 (n=50)	
1 year	56.0±12.7 (n=46)	51.2±15.1 (n=40)	
Change from baseline	9.8±13.7 (n=46)	1.7±6.8 (n=40)	**0.001**
Triglycerides - mg/dl			
Baseline	166.8±104.8 (n=50)	154.7±67.8 (n=50)	

**End point**	**Gastric bypass**	**Medical therapy**	**P value**
1 year	85.7±46.2 (n=46)	130.0±55.0 (n=40)	
Change from baseline	-81.5±105.0 (n=46)	-22.9±70.5 (n=40)	**0.001**†
Uric acid - mg/dl			
Baseline	5.8±1.6 (n=50)	5.7±1.8 (n=50)	
1 year	4.4±1.2 (n=46)	5.4±1.2 (n=40)	
Change from baseline	-1.4±1.3 (n=46)	-0.2±1.9 (n=40)	**0.001**
High-sensitivity C-reactive protein - mg/l			
Baseline	11.5±10.0 (n=50)	10.6±11.2 (n=50)	
1 year	3.1±10.4 (n=46)	8.1±9.3 (n=40)	
Change from baseline	-8.8±15.3 (n=46)	-2.0±7.3 (n=40)	**<0.001**†
10-year framingham risk score - %§			
Baseline	6.9 ± 6.8 (n=50)	6.6 ± 5.7 (n=50)	
1 year	4.5 ± 4 (n=46)	6.9 ± 5.3 (n=39)	
Change from baseline	-2.1 ± 4 (n=46)	0 ± 3.4 (n=39)	**0.003**†
Septum diastolic thickness - mm			
Baseline	9.4 ± 1.2 (n=50)	9.8 ± 1.6 (n=48)	
1 year	8.8 ± 1.1 (n=47)	9.3 ± 1.3 (n=38)	
Change from baseline	-0.6 ± 1.3 (n=47)	-0.4 ± 1.6 (n=37)	0.65†
Ejection fraction - %			
Baseline	67.4 ± 3.6 (n=50)	67.3 ± 4.9 (n=48)	
1 year	67.3 ± 6.6 (n=47)	67.6 ± 3.9 (n=39)	
Change from baseline	0 ± 7.3 (n=47)	-0.1 ± 4.8 (n=37)	0.84†

Plus–minus values are means ±SD, ABMP indicates ambulatory blood pressure monitoring. HOMA-IR indicates homeostatic model assessment- insulin resistance. P-values were estimated by repeated measures ANOVA models adjusted by baseline values, unless indicated otherwise.

* Body-mass index: weight in kilograms divided by the square of the height in meters.

† P-value estimated with generalized estimating equation models.

‡ HOMA-IR index: An indirect measure of insulin resistance calculated from levels of fasting plasma glucose and insulin.

§ 10-year Framingham risk score: estimative of 10-year risk of developing cardiovascular disease by Framingham risk score.

Supplemental Table 4. 24-hour Ambulatory blood pressure monitoring at 12months

End point	Gastric bypass	Medical therapy	Between-group difference, Mean (95% CI)	P value
Awake blood pressure - mm Hg				
Systolic blood pressure	125.4 ± 12.7 (n=48)	125.9 ± 12.2 (n=34)	-0.9 (-6.0 to 4.2)	0.73
Diastolic blood pressure	80.9 ± 12.1 (n=48)	79.5 ± 9.3 (n=34)	1.4 (-3.0 to 5.8)	0.54
Sleep blood pressure - mm Hg				
Systolic blood pressure	112.7 ± 15.0 (n=48)	114.5 ± 12.3 (n=34)	-2.1 (-7.6 to 3.3)	0.44
Diastolic blood pressure	67.7 ± 12.7 (n=48)	67.8 ± 9.2 (n=34)	-0.5 (-4.8 to 3.9)	0.83

Plus–minus values are means ±SD. CI indicates confidence interval. Mean differences between groups, 95% CI and P-values were estimated by repeated measures ANOVA model adjusted by baseline values.

Supplemental Table 5. Nutritional parameters

	Gastric Bypass			Medical Therapy		
	**Baseline**	**12 months**	**P Value***	**Baseline**	**12 months**	**P Value***
Anemia†	6 (3/50)	20 (9/46)	0.01	10 (5/50)	10 (4/40)	0.56
Secondary hyperparathyroidism ‡	13 (6/45)	14 (6/42)	1	NA	NA	NA
Hypovitaminosis B12 §	9 (4/46)	28 (12/43)	0.01	NA	NA	NA
Hypoalbuminemia ‖	2 (1/46)	0 (0/43)	1	NA	NA	NA
Iron deficiency #	0 (0/46)	0 (0/43)	NA	NA	NA	NA
Ferritin deficiency **	2 (1/46)	7 (3/43)	0.56	NA	NA	NA

Values are % (n/N). NA indicates not applicable. These laboratory tests (except for anemia) were performed only in patients who were submitted to gastric bypass.

* All P values were calculated to compare baseline and 12-months using the McNemar Test

† Anemia: hemoglobin levels lower than 12 g/dl in women and lower than 13 g/dl in men

‡ Secondary hyperparathyroidism: parathyroid hormone levels higher than 69 pg/ml.

§ Hypovitaminosis B12: serum B12 levels lower than 193 pg/ml.

‖ Hypoalbuminemia: plasma albumin levels lower than 3.5 g/dl.

Iron deficiency: serum iron levels lower than 49 µg/dl.

** Ferritin deficiency: serum ferritin levels lower than 9 ng/mL in women and lower than 28 ng/ml in men.

Supplemental Table 6. Sensitivity Analyses of Gastric Bypass versus Medical Therapy Effect on the Primary End Point

Sensitivity Analyses	Gastric Bypass	Medical Therapy	Rate ratio (95% CI)	P Value
Complete-case *	85 (41/48)	14 (6/44)	6.3 (3.0 to 13.3)	<0.001
Per protocol †	91 (41/45)	13 (6/47)	7.1 (3.4 to 15.2)	<0.001
As treated ‡	91 (41/45)	12 (6/51)	7.7 (3.6 to 16.5)	<0.001
Worst-case scenario §	82 (41/50)	24 (12/50)	3.4 (2.0 to 5.7)	<0.001
Multivariate model ‖	84 (41/49)	13 (6/47)	6.2 (2.9 to 13.1)	<0.001
Multiple imputation analysis#	85	13	6.5 (3.4 to 12.2)	<0.001

Values are % (n/N)

* Complete cases analysis: analysis without imputation

† Per protocol analysis: excluded patients from gastric bypass group who did not undergo surgery

‡ As treated analysis: patients randomized to gastric bypass group who did not undergo surgery were analyzed in the medical therapy group.

§ Worst-case scenario analysis: in case of primary end point missing it was considered positive in the medical therapy group and negative in the gastric bypass group.

‖ Rate ratio adjusted for body-mass index, number of antihypertensive medications at baseline, 10-year Framingham risk score, basal insulin level at baseline and duration of hypertension.

Analysis was performed with the use of multiple imputation of missing number of medications and blood pressure at 12 months using Gibbs sampling with chained equations method[2]. Variables used as predictors were previous values of the imputed measures, intervention arm, duration of hypertension, age, and

sex. The proportions of patients with positive end point were obtained by the average of the imputed data. Rate ratio was estimated by pooled *Poisson* regression with robust variance.[3]

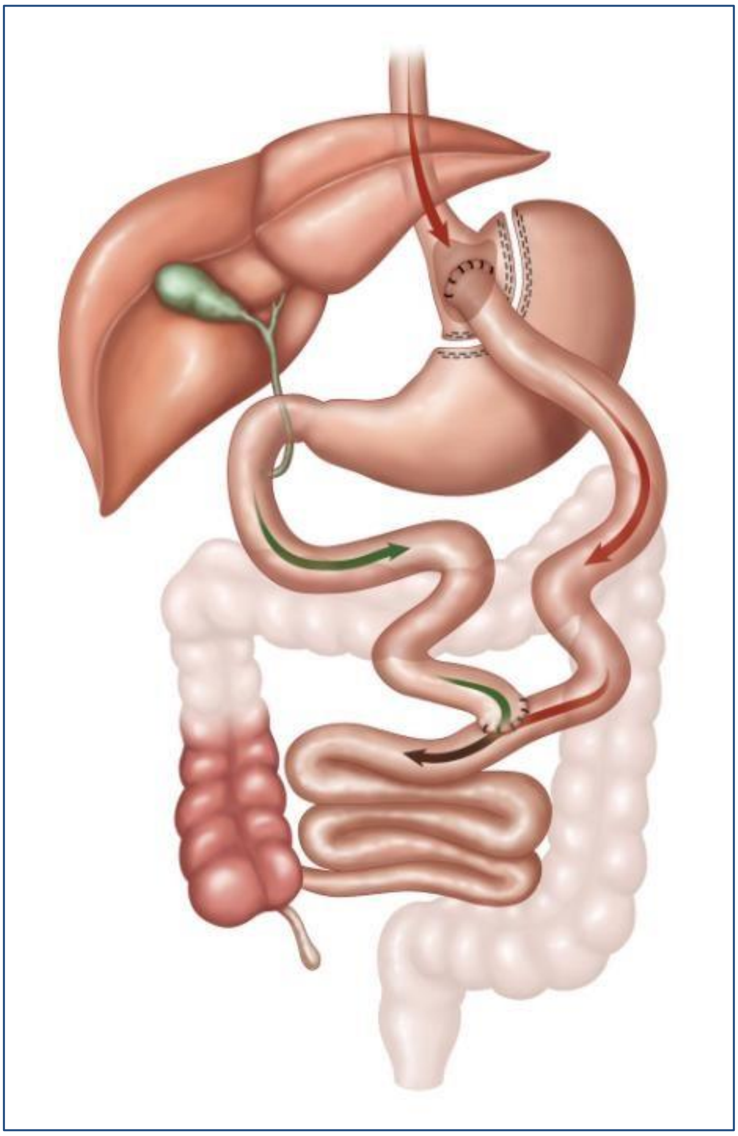

Supplemental Figure 1.

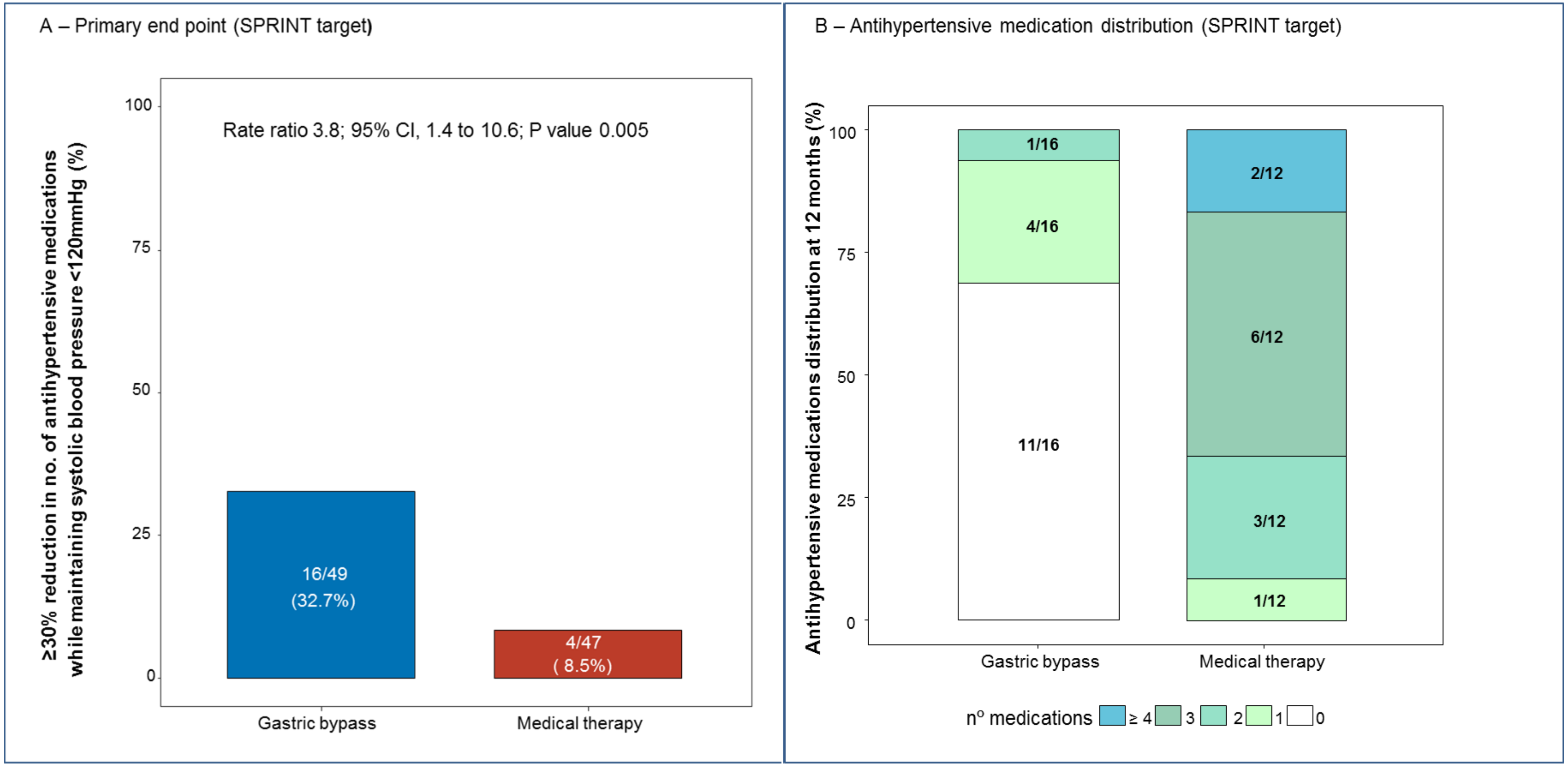

Supplemental Figure 2.

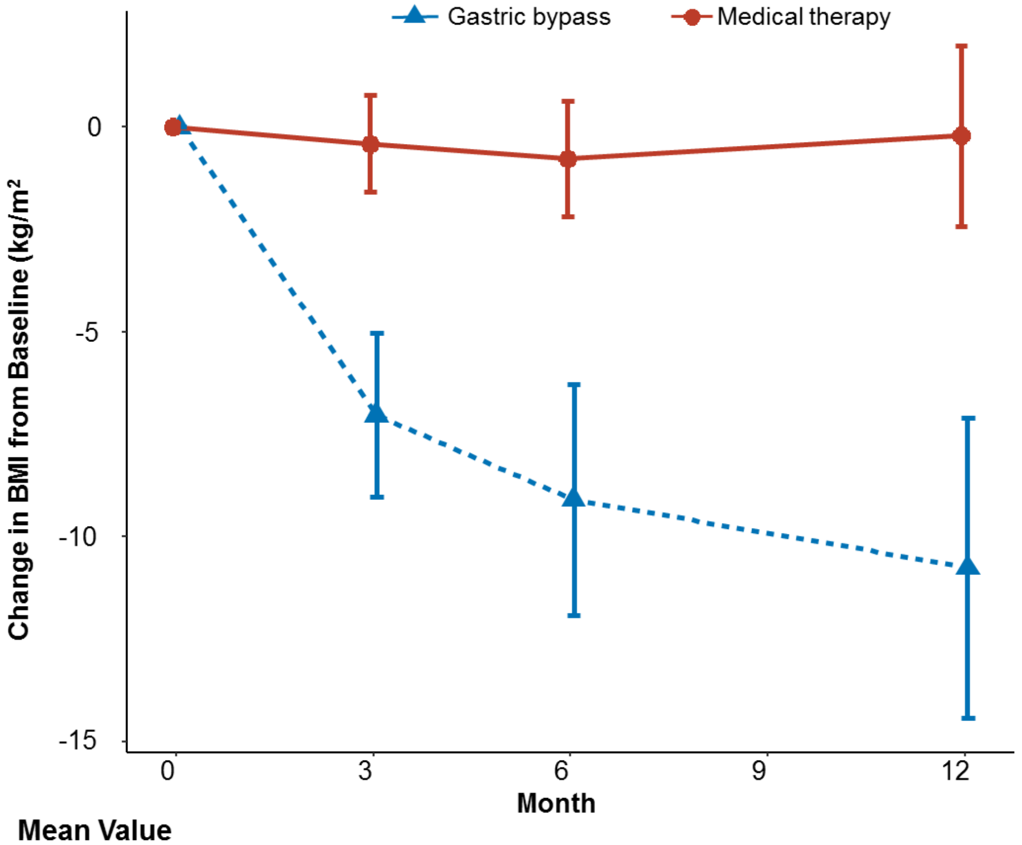

Mean Value at Visit

Gastric bypass	37.4	30.4	28.2	26.8
Medical therapy	36.4	35.9	36.0	36.3

Supplemental Figure 3.

Supplemental Figure titles and legends

Supplemental Figure 1. Roux-en-Y gastric bypass

Supplemental Figure 2. Proportion of patients with reduction of the total antihypertensive drugs of at least 30% while maintaining office systolic blood pressure lower than 120 mm Hg (SPRINT target), Antihypertensive medication distribution.

This post hoc analysis assesses the effect of gastric bypass versus medical treatment on the reduction of the total antihypertensive drugs while keeping office systolic blood pressure lower than 120 mm Hg, as targeted in The Systolic Blood Pressure Intervention Trial (SPRINT)[4] (**A**), the distribution of number of antihypertensive medications use at 12 months in patients with office systolic blood pressure lower than 120 mm Hg, according to SPRINT levels (**B**).

Supplemental Figure 3. Body-mass index

Change from baseline of body-mass index (BMI, the weight in kilograms divided by the square of the height in meters) was plotted at 3, 6, and 12 months. I bars indicate standard deviation. Mean values in each group are provided below the graphs.

References

1. Sociedade Brasileira de Cardiologia (SBC); Sociedade Brasileira de Hipertensão (SBH); Sociedade Brasileira de Nefrologia (SBN). V Guidelines for ambulatory blood pressure monitoring (ABPM) and III Guidelines for home blood pressure monitoring (HBPM). *Arq Bras Cardiol*. 2011; 97:1-24.
2. Van Buuren, S., Groothuis-Oudshoorn, K. mice: Multivariate Imputation by Chained Equations in R. *Journal of Statistical Software*. 2011; 45:1-67.
3. Barnard, J, Rubin, DB. Small sample degrees of freedom with multiple imputation. *Biometrika*, 1999; 86:948-955.
4. The SPRINT Research Group. A Randomized Trial of Intensive versus Standard Blood-Pressure Control. *N Engl J Med*. 2015;373:2103–2116.
